# Control of quiescence and activation of human muscle stem cells by cytokines

**DOI:** 10.1371/journal.pone.0327701

**Published:** 2025-12-05

**Authors:** Katharine Striedinger, Emilie Barruet, Elena Atamaniauc, Karla Linquist, Chris Knott, Andrew Brack, Jason H. Pomerantz

**Affiliations:** 1 Program in Craniofacial Biology, Eli and Edythe Broad Center of Regeneration Medicine, University of California San Francisco, San Francisco, United States of America; 2 University of California San Francisco, San Francisco, United States of America; 3 Division of Plastic and Reconstructive Surgery, Department of Surgery, and Department of Orofacial Sciences, University of California San Francisco, San Francisco, United States of America; UC Los Angeles: University of California Los Angeles, UNITED STATES OF AMERICA

## Abstract

Skeletal muscle homeostasis and repair depend on the activation of tissue resident stem cells called satellite cells. To understand the early molecular basis of human satellite cell activation, epigenomics, transcriptomics and protein analysis were performed in quiescent and experimentally activated human satellite cells. Cytokine signaling pathways were enriched in activated human satellite cells revealing high cytokine enrichment, including CCL2, CCL20, CXCL8, IL-6, TNFRSF12A, ILR1, CSF-1 and FGF2. Functional roles of these observed changes are supported by *in vivo* experiments showing that chemokine inhibitors increase engraftment and regeneration capacity of human satellite cells xenotransplants. Cytokines, chemokines and associated signaling pathways in the early stages of human satellite cell activation may underlie disparate muscle responses in neuromuscular inflammatory and degenerative disorders and consequently are potential entry points for clinical applications towards muscle repair.

## Introduction

Skeletal muscle resident stem cells called satellite cells (MuSC), ensure muscle tissue homeostasis, repair and regeneration throughout life [1–3]. Quiescent and self-renewing satellite cells in mice and humans are characterized by the expression of the transcription factor Paired Box Gene 7 (PAX7), crucial for the development and regeneration of skeletal muscle by regulating MuSC [4]. In homeostatic conditions, specific signals maintain MuSC in a quiescent state. But upon muscle injury, MuSC transition from a GO state to rapid activation and cell cycle re-entry (S phase), accompanied by orchestrated temporal, and spatial activation signals causing MuSC to express myogenic regulatory factors: myogenic factor 5 (MYF5), myogenic differentiation 1 (MYOD1), MYOGENIN (MYOG) and progressive loss of PAX7 expression [1–5]. This leads to asymmetric divisions towards differentiation into muscle progenitor cells or myoblasts, migration, and fusion into multinucleated myofibers to regenerate muscle or symmetric self-renewal divisions for niche maintenance [6,7].

While recent experiments in animal models have provided information about signaling mechanisms in the muscle niche [4,6,8] much of the environmental cuing that governs early MuSC activation remains less well understood, especially in humans. Furthermore, study of impairment of satellite cell function that occurs in most neuromuscular disorders found that almost half of the known myopathogenes (genes altered in neuromuscular disorders) are differentially expressed during the initial hours of satellite cell activation [9]. A majority of these myopathogenes directly respond to PAX7 and MYOG [9].

We recognized the importance of studying gene expression changes associated with the early activation process of human muscle satellite cells (HuMuSC). This required development of an experimental model of HuMuSC activation allowing for high throughput epigenomic characterization of activated compared to quiescent HuMuSC.

By modeling HuMuSC activation in a reproducible experimental context, we found that in a short time window, HuMuSC undergo considerable epigenetic and transcriptomic changes. Among the most prominent groups of genes to change during early HuMuSC activation is cytokine signaling. Cytokines in satellite cells regulate quiescence and self-renewal, and chemokines (chemotactic cytokines) are involved in cell migration and proliferation in response to muscle damage [10]. We hypothesized that specific cytokines regulate quiescence and activation in human satellite cells. Here, Our goal here was to examine the epigenetic events of cytokine alterations upon HuMuSC activation, to present novel observations of specific chemokine activity in activated HuMuSC along with experimental evaluation of functional importance in muscle regeneration.

## Results

### Experimental model of HuMuSC activation and associated epigenomic changes

**Fig 1:**
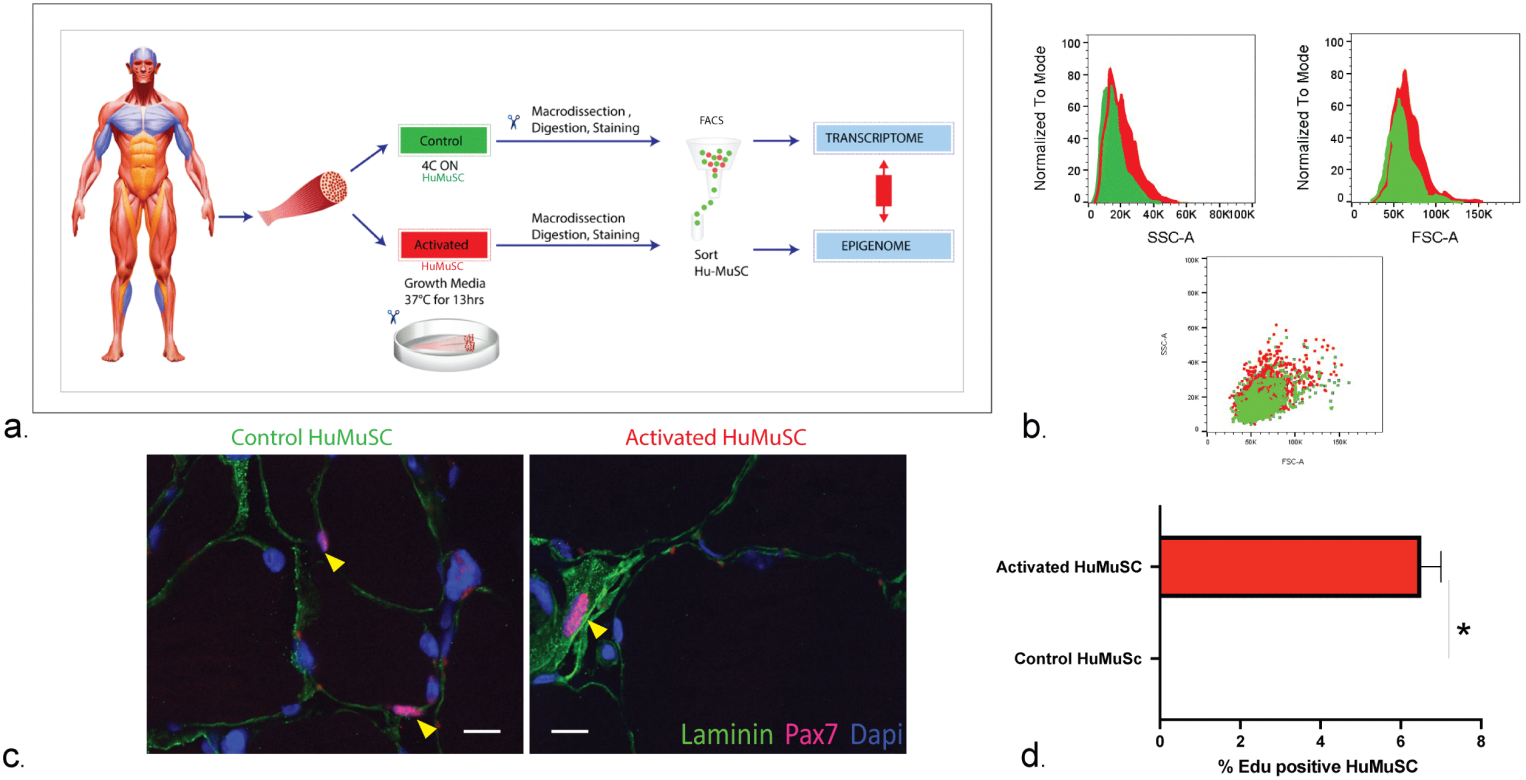
Model of human satellite cell activation. **(a)** After collection, human muscle biopsies were either kept at 4ºC overnight until dissociation (Control) or immediately dissociated and incubated with growth media at 37ºC overnight until further mechanical and enzymatic dissociation (Experimentally stimulated). Then, human muscle satellite cells (HuMuSC) were sorted for CXCR4 + /CD29 + /CD56 + using fluorescence activated cell sorting (FACS) and used in downstream experiments, for example single cell transcriptomics and epigenomics. Typical isolation was 10,000 HuMuSC per gram of human muscle and 68 biopsies were used with a mean of n = 38,500 cells isolated per sort. **(b)** An increased Side (SSC-A) and Forward (FSC-A) scatter in the stimulated HuMuSC (red) compared to of control HuMuSC (green) show increased cell size and granularity when the HuMuSC undergo activation. **(c)** HuMuSC from *in situ* activated human muscle show an increase in size (yellow arrowhead) identified with PAX7 (red) immunostaining compared to control muscle HuMuSC. Scale bar: 10 uM. **(d)** Higher percentage of EdU labeled HuMuSC in the activated muscle (6.5 ± 0.2% n = 3) compared to non-labeled non dividing quiescent control HuMuSC 24 hrs after FACS sorting.

Acknowledging that various muscle stimuli and injuries may elicit differing satellite cell activation responses, we sought to develop an experimental model of HuMuSC activation that would reasonably mimic common natural activation characteristics and would be reproducible. Such a model would be expected to induce some or all the canonical changes previously described to occur with satellite cell activation. The activation model described here uses a combination of mechanical injury, ambient temperature and Fibroblast Growth Factor 2 (FGF2) enriched growth media to activate freshly isolated HuMuSC (Fig 1a).

**Fig 2:**
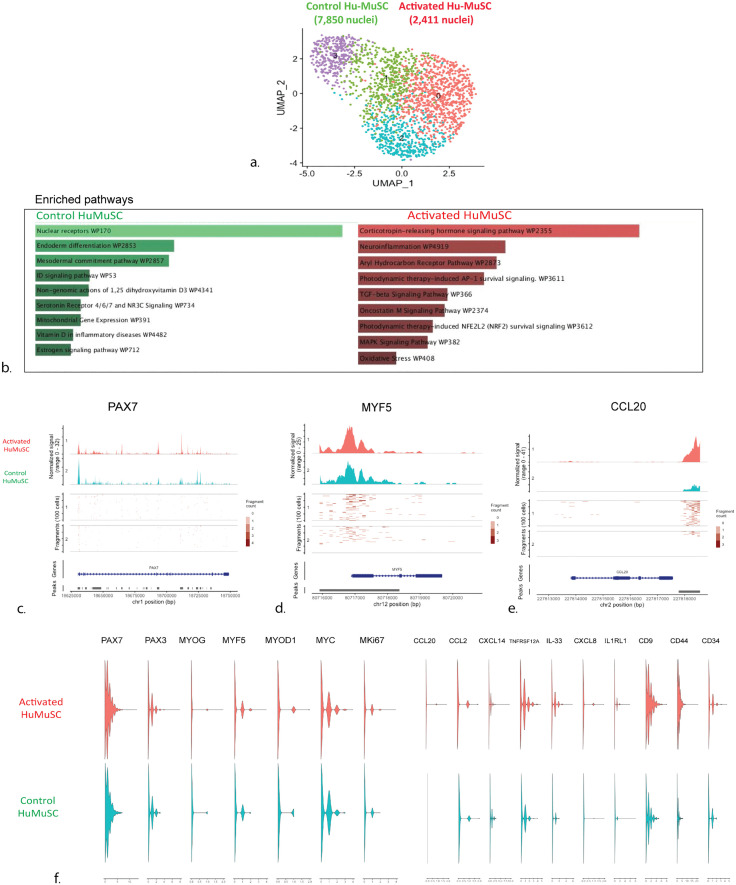
Single-nuclei chromatin accessibility assay (snATAC) in control and experimentally stimulated human satellite cells from the vastus muscle of a 56-year-old male. **(a)** UMAP representations of snATAC-seq of control and HuMuSC. Individual dots represent single cells, in total 9,232 HuMuSC nuclei, distributed in 7,850 from HuMuSC, and 2,411 from control. A clear epigenetic shift distinguishing control from HuMuSC becomes apparent in the resulting four HuMuSC clusters (resolution of 0.8). **(b)** Top differentially expressed pathways based on the highest differential peak expression between control and activated HuMuSC sorted by p-value using WikiPathways [38]. Note the TGF-B, Oncostatin-M and MAPK, among the other listed pathways that are significantly enriched in the activated HuMuSC. **(c-f)** Violin plots showing the distribution of chromatin fragments using scATAC, on activated HuMuSC and control. Open chromatin violin plots showing presence of PAX7 (**c**) in both HuMuSC and controls while increased fragments of Myogenic factors like MYF5 **(d)**, MYOG, MYC and MYD1(**f**) in the HuMuSC compared to controls. **(e,f)** Increased distribution and frequency of chemokine open chromatin fragments including CCL20 **(e)**, CCL2, IL-33 in HuMuSC compared to controls **(f).** These results show significant epigenetic cytokine peaks during activation, preceding overall changes in muscle stem cell transcription factors and myogenic regulatory genes in human satellite cells.

**Fig 3:**
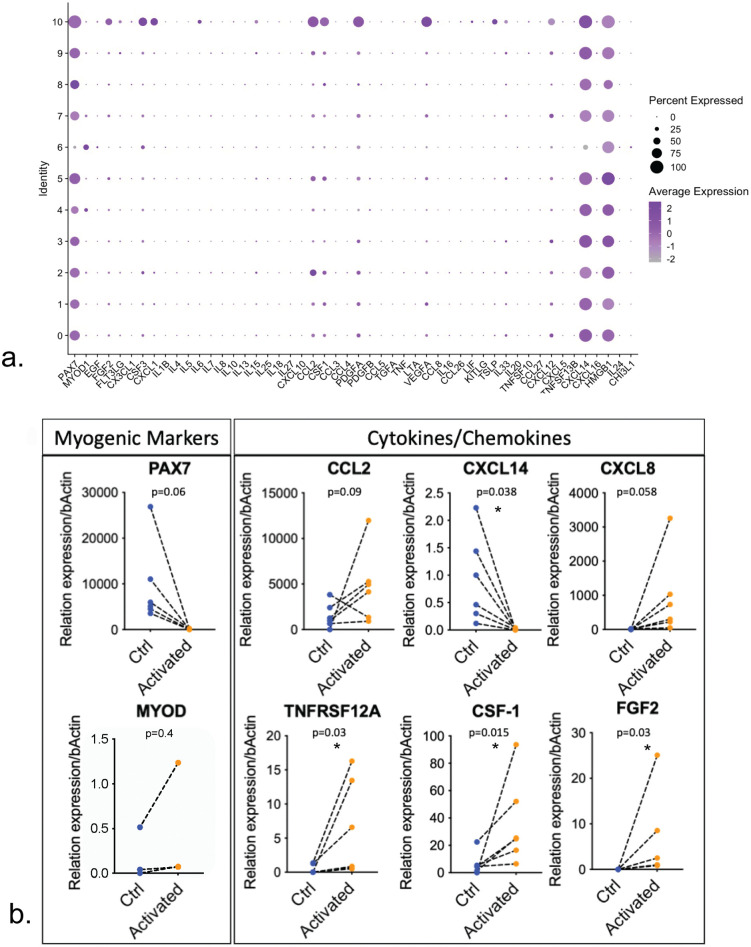
Differential temporal expression of cytokines and chemokine ligands and receptors during human satellite cell activation. **(a)** We evaluated cytokine and chemokine transcription of HuMuSC from twelve different donors, and three different muscles: vastus lateralis, pectoralis major and rectus abdominis. As expected, the majority of HuMuSC in these samples express high levels of *PAX7* transcripts and low levels of *MYOD1*. Cytokine transcripts *CCL2, CXCL14* and *HMGB1* are abundantly expressed among all HuMuSC clusters from unstimulated muscle. Cluster 10 exhibits the highest percentage of HuMuSC expressing cytokines, chemokines transcripts and growth factors, including *FGF2, CSF3, CXCL1, CCL2, CSF1, PDGFA, VEGFA, CXCL12*. **(b)** To further evaluate cytokine expression during satellite cell activation we compared cytokine transcripts expression using RT-PCR in control and activated HuMuSC (HuMuSC). Each paired control and activated muscle from the same donor are depicted with a line. Consistent with our epigenomic findings, there is a tendency of *PAX7* transcripts to be rapidly downregulated after activation, and elevated levels of *MYOD1* upon activation. Chemokines *CCL2 and CCL8* are upregulated in the HuMuSC, as are TNFRSF12A, CSF-1 and FGF2 in the HuMuSC compared to controls. In contrast CXCL14 was found significantly downregulated in HuMuSC compared to controls. Each pair of observations connected with a dotted line was derived from an independent human muscle sample n = 3-6. **(**Wilcoxon test *P*- values are * < 0.05).

**Fig 4:**
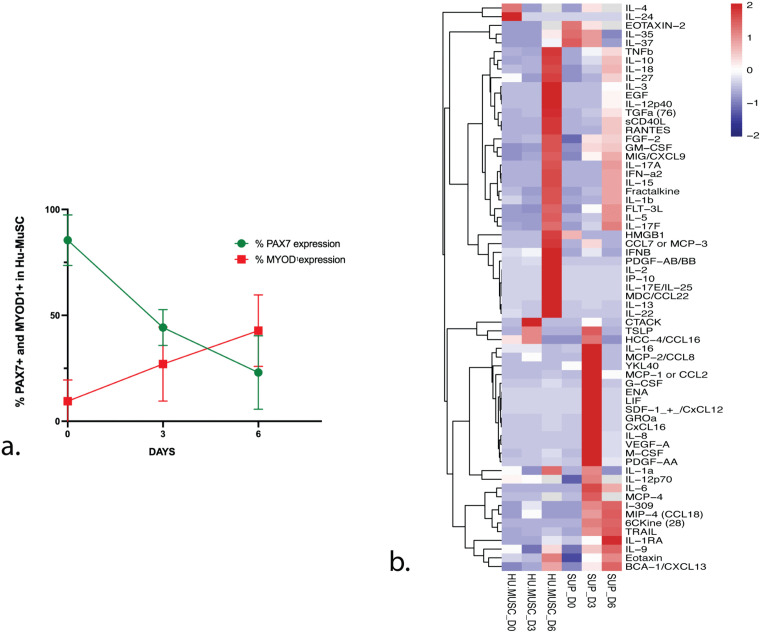
The expression of PAX7 and MYOD1 correlate with differential expression of diverse chemokines in HuMuSC. **(a)** Quantification of protein expression for PAX7+ and MYOD1 positive HuMuSC at day 0, 3 and 6 in vitro, showing an inverse correlation as progressively PAX7 expression decreases and MYOD1 expression increases in HuMuSC as they activate *in vitro*. Using linear regression PAX7 slope varies significantly p = 0.0001 and MYOD1 slope also varies significantly p = 0.05 n = 3 **(b)** Heat map displaying the expression levels of all cytokine protein detected in HuMuSC from collection as they activate progressively at day 0, day 3 and day 6 *in vitro*, using the high throughput proteomic Multiplex assay. Out of 93 screened 76 cytokines were identified in the supernatant of HuMuSC, with the highest concentrations including CXCL8, CCL2, GROa and M-CSF. Note the sequential expression of cytokines at different time points, with early expression at day 0 of IL-4 and IL-24 and in the IL-35, IL-37 and Eotaxin-2 in the supernatant at day 0. Later the peak of expression in the supernatant of CCL2, CCL8, LIF, CXCL8, IL-6 occur at day 3 *in vitro* and other cytokines are primarily expressed later at day 6 in both HuMuSC lysates and supernatant n = 3.

Activated HuMuSC were increased in size as shown in the forward and side scatter images compared to controls (Fig 1b). High magnification images of the control and activated myofiber fragments also show increased size of the PAX7 immunolabeled HuMuSC (Fig 1c). Only activated HuMuSC, were positive for Edu (6.5 ± 0.2%) within the first 8 hrs after sorting compared to controls (Fig 1d). Activated HuMuSC and control HuMuSC express different levels of PAX7 and MYOD1 expression after FACS isolation at the epigenomic (Fig 2f), transcriptomic (Fig 3a,b) and protein levels (Fig 4a) confirming these are satellite cells in early stages of activation.

To understand molecular epigenetic modifications that may control the observed cellular changes of early activation, we used the single cell Assay for Transposase Accessible Chromatin (scATAC) to compare the open chromatin regions, the significant peaks, and the cellular pathways differentially expressed in control HuMuSC and activated HuMuSC (Fig 2). The resulting dataset after quality control included 9,232 nuclei distributed in 7,850 from activated HuMuSC and 2,411 from the control HuMuSC (Fig 2a). Counterintuitively, the median fragments per cell were higher in the control HuMuSC (14,631) compared to the activated HuMuSC (5,713). This observation indicates a high level of open chromatin in control HuMuSC.

After data normalization and linear dimensional reduction, we constructed a low-dimensional visualization of the DNA accessibility assay using uniform manifold approximation and projection (UMAP). The UMAP shows a clear shift in chromatin conformation upon HuMuSC activation (Fig 2a), distinguishing control clusters (mainly distributed in cluster 1 and cluster 3) from the activated HuMuSC, represented mostly in clusters 0 and 2 (Fig 2a).

We identified significantly differentially expressed peaks (p values < 0.05) between control and activated HuMuSC. We identified 47 significantly different genomic accessible peaks from which 10 corresponded to non-coding RNA. Remarkably, 8 of 37 coding regions, corresponded to known cytokines, chemokines, immune system mediators and growth factors. We then performed cellular pathway analysis based on the enriched peaks and open chromatin regions in control and activated HuMuSC. Genome wide pathway analysis revealed increased activity of interleukin and immune system pathways in activated HuMuSC. (Fig 2b). While the control HuMuSC enrichment peaks and corresponding pathways is on nuclear differentiation and mitochondrial gene expression pathways, the activated HuMuSC exhibit enrichment of corticotropin releasing hormone, neuroinflammation, TGF-beta signaling, Oncostatin and MAPK signaling pathways (Fig 2b).

PAX7 open chromatin was present in both stimulated and control samples, but upon stimulation, PAX7 open chromatin first peaks decrease, especially at the transcription start site (TSS) region of the PAX7 gene (Fig 2c), while MYF5 transcription factor open chromatin peaks increase in the activated HuMuSC (Fig 2d).

Since many of the altered cellular pathways involve cytokines and their effectors, we decided to further investigate the most significant changes. The Monocyte chemoattractant protein-1 (CCL2) and the CXCL8 have higher peaks in activated HuMuSC compared to paired controls (Fig 2e,f). MKi67 has more open chromatin fragments in activated HuMuSC compared to controls (Fig 2f).

HuMuSC ATAC data reveals that inflammatory cytokines including IL-33 and CXCL8 (Fig 2f), and the receptors TNFRSF12A and IL1RL1, as well as the antigen receptors CD14, CD44, CD9 increase upon stimulation (Fig 2f). These findings demonstrate a broad engagement of the known cytokine, chemokine and immune modulating pathways immediately after HuMuSC stimulation.

### Cytokine transcriptome changes upon HuMuSC activation

The open chromatin results prompted us to confirm gene expression using analysis of single cell RNA from freshy isolated HuMuSC. We have previously shown that HuMuSC from healthy muscle biopsies exist in distinct transcriptional clusters that differ in their gene expression profiles [11]. Within a specific muscle sample, the majority of HuMuSC express patterns consistent with quiescence, but have distinct expression profiles of unclear significance, and some express genes associated with activation [11].We evaluated cytokine and chemokine transcription of HuMuSC from 12 human muscle samples. As expected, the majority of HuMuSC in these samples express high levels of *PAX7* transcripts and lower levels of *MYOD1* HuMuSC (Fig 3a). Cytokine transcripts such as, *CCL2, CXCL14* and *HMGB1* are abundantly expressed among all HuMuSC clusters (Fig 3a). Cluster 10 exhibits the highest percentage of HuMuSC expressing cytokines, chemokines transcripts and growth factors, including *FGF2, CSF3, CXCL1, CCL2, CSF1, PDGFA, VEGFA, CXCL12* (Fig 3a).

To further evaluate cytokine expression during satellite cell activation we used RT-PCR from paired control and activated HuMuSC. Each pair comes from a single muscle donor (Fig 3b). Consistent with our epigenomic findings, *PAX7* transcripts are rapidly downregulated after activation, giving rise to elevated levels of *MYOD1* upon activation (Fig 3b).

There is a tendency for some chemokines including *CCL2* and *CXCL8* to be upregulated in HuMuSC compared to controls, and a statistically significant increase of *TNRSF12A*, *CSF-1* and *FGF2* in activated HuMuSC compared to controls (Fig 3b). These quantitative changes in cytokine transcripts in activated HuMuSC were concordant with the open chromatin data with the exception of *CXCL14* which wsa significantly downregulated upon activation (Fig 3b).

To further characterize the activation state of HuMuSC in this assay, protein expression was evaluated by immunostaining. The percentage of PAX7+ and MYOD1 + HuMuSC *in vitro* over the first 6 days was quantified. PAX7 significantly decreased to less than half in the first 3 days while MYOD1 significantly and progressively increased over 6 days (Fig 4a), confirming that this assay captures the early temporal window of HuMuSC activation and progression towards differentiation.

To investigate cytokine protein levels during culture activation, we used a high throughput proteomic multiplex assay to identify the cytokine ligands and receptors expressed in HuMuSC from three different muscle samples. With the Human Cytokine/Chemokine Array (Eve technologies), we tested 92 different cytokines in the supernatant and in the cellular lysates of HuMuSC collected at day 0, at day 3 and at day 6 after HuMuSC were plated under growth conditions. After background subtraction analysis, 35 cytokines were found to be expressed in HuMuSC, with varying concentrations during activation in culture (Fig 4b).

The cytokine protein concentration in cell lysates and supernatant is represented in a Heat map (Fig 4b) displaying the expression levels at different times points. The earliest expressed cytokines were IL-4 and IL-24, detected in lysates at day 0 while Eotaxin-2, IL-35, IL-37 were the earliest detected cytokines in the supernatant (Fig 4b). The expression of CXCL8, YKl40, CCL2, GROa and M-CSF is apparent on day 3 in supernatant. Ten more cytokines were also detected in the cellular lysates, including IL-4, IL-24 and CCL16 as early as day 0 (Fig 4b). Noticeable, CCL2, CXCL8 and IL-6 show highest expression in Supernatant at day 3. We observed the maximum expression of TGF-B, CCL7, HMGB1 in HuMuSC lysates at day 6. A singular cytokine, HMGB1, was found to be expressed in HuMuSC at higher concentrations than all the other cytokines (Fig 4b). The expression of PAX7 and MYOD1 correlate with a differential expression of diverse cytokines and chemokines in HuMuSC (Fig 4a,b).

### Chemokine gain and loss of function modulates engraftment after human satellite cell xenotransplantation

Our expression data suggest that chemokines may play a functional role during HuMuSC activation. Moreover, satellite cell activation and muscle repair involve migration from the niche at the basement membrane to the center of the myofiber to initiate myofiber regeneration. This migration could be expected to be controlled by chemokine activity. We identified CCL2 as one of the most abundantly present chemokine transcripts in HuMuSC and found that it is actively upregulated during early activation. Thus, CCL2 was selected as a candidate target to explore the effect of gain and loss of function on engraftment and myofiber formation by HuMuSC *in vivo*.

**Fig 5:**
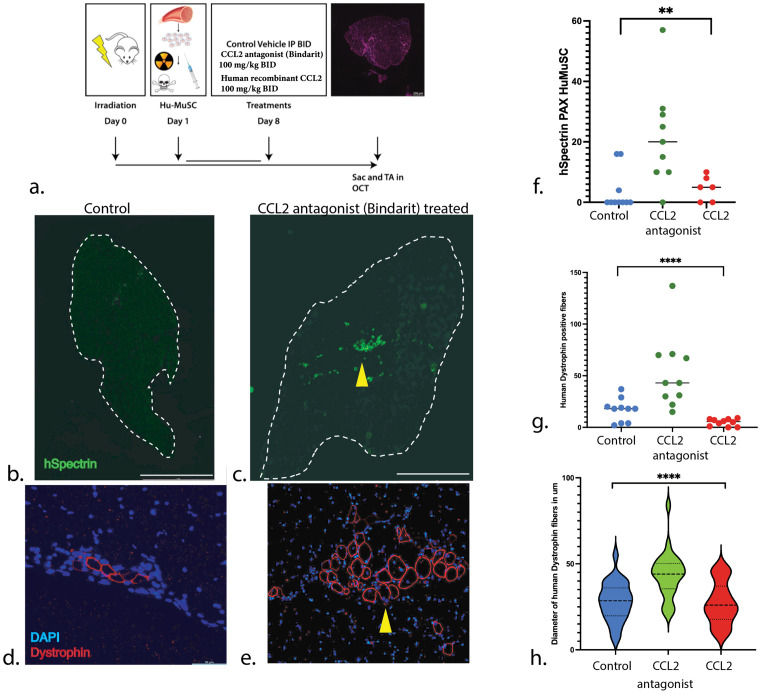
Xenotrasplants of HuMuSC in TA of mice. **(a)** Model for xenotransplants of HuMuSC in the TA of NSG mice, treated with either the CCL2 antagonist (Bindarit) or a human recombinant CCL2 (from Pepotech), or vehicle control. **(b-e)** Sections of TA showing a representative example of Control **(b,d)**, Bindarit (**c,e**) treated mice. By means of immunostaining, we observe a high number of human spectrin **(b,c,f)** and Dystrophin positive fibers **(d,e,g)** in the Bindarit treated muscle (yellow arrowheads) compared to controls **(g)**. The diameter of the newly formed human fibers is significantly higher in the Bindarit treated compared to controls **(h)**. n = 10 mice for each group. Scale bar represents (**b, c**: 1 mm); (**d, e**: 50 μm) Statistical analysis was performed using paired Student’s *t-*tests; *P*- values are * < 0.05, *** < 0.001.

We treated immunodeficient mice with either CCL2 antagonist (Bindarit) or a human recombinant CCL2 (from Pepotech) or vehicle controls daily for 7 days after HuMuSC transplantation in the Tibialis Anterior (TA) (Fig 5a). The effect of Bindarit is thought to be mediated by the downregulation of the NF-κB pathway [12,13] and TGF-B signaling, inhibiting CCL2, and also affecting CCL7 and CCL8 [12]. Three weeks after transplantation of 2,000 HuMuSC, the TAs were collected and the number and diameter of the human Dystrophin positive fibers as well as the number of xenotransplanted HuMuSC expressing PAX7 + were quantified: a significantly higher number of human Spectrin (Fig 5b,5c,5f) and Dystrophin positive fibers (Fig 5d,5e) were observed in the Bindarit treated muscle compared to controls (Fig 5g). The diameter of the newly formed human fibers is also significantly higher in the Bindarit treated group compared to controls (Fig 5h). These results show a more robust cluster and higher number of human fibers in the Bindarit treated group compared to controls and CCL2 agonist (Fig 5b–5h) suggesting that Bindarit promotes differentiation and associated engraftment of HuMuSC.

Co-Immunostaining of PAX7 and human Spectrin were used to identify undifferentiated HuMuSC. While in controls HuMuSC are sparse as in prior studies [14,15] in quiescent muscle, in the Bindarit treated group there were more frequent HuMuSC PAX7+ and hSpectrin+ cells, suggesting that HuMuSC survive, returned to quiescence and/or self-renew more readily in a chemokine-depleted environment (Fig 5f). These results support the idea that reducing the levels of (CCL2, CCL7 and CCL8) in the host muscular niche by means of exogenous agents, can result in higher engraftment after transplantation and greater myofiber formation capacity (**Fig 5a**–**5h**).

## Discussion

In this study, we examined the epigenetic events of the transition of human satellite cells from quiescent stem cells, into activated proliferating muscle-stem cell progenitors, a process essential for proper muscle maintenance and regeneration. Our findings identify a complex network of cytokines, chemokines, ligands, and associated pathways acting simultaneously during the early activation of human satellite cells.

Our experimental model of early HuMuSC activation recapitulates previously described changes of activation, including increase in size, granularity and EdU uptake [4,16]. It has been described that the low metabolism of quiescent MuSCs is primarily dependent on mitochondrial fatty acid oxidation and oxidative phosphorylation to promote epigenetic modifications that repress myogenic transcription programs [4] and conserve the stemness of MuSC. In contrast, activated MuSC display a different metabolic phenotype as they increase in cell size and shift toward anaerobic glycolysis [4]. This metabolic environment of activation allows for rapid biosynthesis, supporting growth and proliferation.

Despite the metabolic shift during activation, our single cell open chromatin data showed that the median fragments per cell were almost three times higher in the control HuMuSC compared to the experimentally stimulated HuMuSC, in support of the concept that quiescence is not a passive process but a highly transcriptionally active cellular state under tight regulation to decrease cellular stress and ensure long term maintenance [16–18]. This is consistent with chromatin data studies in MuSC [19], where the chromatin of quiescent MuSC is largely permissive and becomes more restricted due to increased H3K27 upon MuSC activation, yielding a landscape with many genes enriched in quiescent MuSC but rapidly downregulated upon activation [19].

MuSC quiescence is associated with PAX7 expression and inhibition of MYF5 and MYOD1, which in turn are recognized hallmarks of MuSC activation and myogenic commitment [4]. The early tissue activation model for satellite cells used here allowed observation of some of these earliest changes. Accordingly, we confirmed the expected observation that human unstimulated satellite cells have higher *PAX7* open chromatin fragments than stimulated HuMuSC but stimulated HuMuSC also contain abundant *PAX7* fragments while expressing higher levels of *MYF5, MYOD1* and *MYOG* than controls. *MYF5* is one of the first myogenic regulatory factors to increase with early satellite cell activation [20]. These epigenetic events were detected at the single cell level in this experimental system before detectable changes in other myogenic regulatory factors such as *MYC* and later on *MHC* take place, which characterize states further downstream in myogenic commitment, differentiation and myofiber fusion [4,20].

Studies in mice and humans have identified at least two major subgroups of satellite cells by single cell RNA-seq [4,11,21]. MuSC close to quiescence express high levels of *PAX7*, *Hes1*, *Col2A*1 and genes related to cell cycle arrest and stress resistance, while MuSC in early activation express *MYOD1*, decreased *PAX7* expression and enrichment of genes involved with protein synthesis [21]. Although almost 100% of HuMuSC express PAX7 after isolation from adult human skeletal muscle [22], they rapidly lose PAX7 expression over next 3 days *in vitro*, while increasing expression of MYOD1, mimicking the activation of satellite cells towards myoblasts [22]. This marks a short window of quiescence for HuMuSC *ex vivo* in which we identified extensive changes in cytokine and chemokines transcripts and protein expression. The increase in open chromatin configuration of cytokines in our scATAC experiments correlate with most of the changes in the myogenic regulatory factors during activation of HuMuSC. According to our single cell chromatin data, chemokines are all significantly enriched in stimulated human satellite cells. Therefore, cytokines are one of the earliest groups of genes to change in the activation of HuMuSC and likely do not depend on the myogenic regulatory factor cascade to do so. These findings imply that important regulators and effectors of MuSC behavior act in parallel and possibly independent of canonical myogenic pathways during activation and regeneration.

As HuMuSC become activated, the chromatin regions of cytokine *TNFRSF12A* become highly exposed. TNFRSF12A and the TNF receptor superfamily member fibroblast growth factor-inducible 14 (Fn14), have emerged recently as a pivotal axis for shaping both physiological and pathological muscle responses to acute or chronic injury and disease and in regulating skeletal muscle mass and metabolism [20,23,24]. TNFRSF12A expression results in enhanced proliferation of mouse muscle myoblasts [23]. TNFRSF12A also inhibits satellite cell self-renewal by inhibiting Notch signaling and stimulates the mitogen-activated protein kinase (MAPK) and canonical and non-canonical nuclear factor-kappa B (NF-κB) signaling pathways in myoblasts [20]. FGF (fibroblast growth factors) and MAPK (mitogen-activated protein kinase) signaling pathways also regulate MuSC proliferation and myogenic commitment. FGF signaling from myofibers, satellite cells and fibroblasts controls differentiation, whereas p38 MAPK has been implicated in both self-renewal and differentiation. Our human chromatin supports these findings in mice as a conserved pathway in human muscle, as we also observed TWEAK receptor, MAPK, NF-κB and cytokine signaling pathways enriched in stimulated human satellite cells.

Several transcription factors, cytokines and ligands previously found to have roles in muscle stem cell activation and repair were identified in our experiments in human cells. KLF10, an effector of TGF beta signaling has a functional role in proliferation and differentiation of a variety of tissues, and loss of KLF10 in normal and dystrophic mouse skeletal muscle results in increased fibrosis and decreased grip strength [25]. IL-6 and KLF15 were among the predicted upstream regulators for later activation in a bulk chromatin accessibility study comparing human cultured myoblasts to satellite cells [26]. In animal studies, cytokines that have been described to play active roles in quiescence include the cytokine CXCL14, an endogenous inhibitor of myoblast differentiation and skeletal muscle regeneration [27]. The CXCL12–CXCR4 axis is known to maintain stem cell quiescence and CXCL14 depletion has been implicated in cell cycle withdrawal and acceleration of myogenesis [27]. Oncostatin M (OSM), a member of the interleukin-6 family of cytokines, is a potent inducer of murine muscle stem cell quiescence acting though the hippo pathway to stimulate self-renewal [28]. Our human epigenetics data shows that Oncostatin M pathways are enriched in stimulated human satellite cells.

Several inflammatory myopathies have been associated with CCL2, MCP-3 and RANTES [29–31], and CXCL12 and CCL2 have been shown to be increased in idiopathic inflammatory myopathies [32]. CCL2, MCP-3, RANTES, CXCL14 and CCL2 were all differentially expressed in the first 72 hrs in HuMuSC. CCL2 is a key chemokine implicated in the regulation of migration and infiltration of monocytes/macrophages, and together these findings support the utility experimental model for studying inflammatory myopathy.

Bindarit was chosen because it causes dose dependent selective inhibition of monocyte chemotactic proteins CCL2, CCL7, CCL8 and has anti-inflammatory efficacy in animal disease models *in vivo*. Bindarit modulates cancer-cell proliferation and migration, mainly through negative regulation of TGF-β, AKT signaling and the NF-κB pathway. Our *in vivo* xenograft experiments show that by decreasing CCL2, CCL7 and CCL8 activity with Bindarit, transplanted HuMuSC develop more and larger regenerated human myofibers. These data indicate that the engraftment of HuMuSC and myofiber formation is enhanced by transient inhibition of inflammatory cytokines. Our observations are consistent with a recent study showing that inhibition of inflammatory-induced activation of CCL2 and CCR2 signaling after injury enhances regeneration and functional recovery [33]. Our findings suggest that at least part of the mechanism underlying that observation involves satellite cell mediated muscle formation.

There is evidence of chronic activation of aged satellite cells in mice [34], suggesting that senescence cellular programs involve inflammatory cytokines [35]. It is also known that a chronic inflammatory milieu (high IL-6, TNF-α, interleukin-1 (IL-1), and C-reactive protein “CRP”) is correlated with sarcopenia in aging [36]. These cytokine profiles are thought to lead to a predisposition to age-related sarcopenia, ultimately leading to exhaustion of the PAX7 + satellite cells niche [36,37]. Our data shows that most of the inflammatory cytokines including IL-6, IL-33, CXCL8, TNFRSF12A and chemokines CCL2, CCL7, CCL8, were upregulated in stimulated HuMuSC during activation. This suggests that similar gene expression changes that occur with acute injury and activation occur perhaps in a deregulated fashion in human muscle diseases and aging.

In conclusion, the identification and characterization of HuMuSC cytokine signaling pathways early during the activation process was enabled using an experimental model of ex vivo stimulation. This also permitted functional experiments that suggest importance of these changes in muscle repair and regeneration. The findings of this study using experimental stimulation of HuMuSC will require in vivo, in situ corroboration, since the experimental process of removing muscle tissue and preparation for experimental manipulation likely alters gene expression to some extent before and after activation. Nonetheless, the observation that experimentally stimulated HuMuSC recapitulate the phenotypic changes of activated HuMuSC in vivo support utility of this approach as a faithful model of early HuMuSC activation.

## Materials and methods

### Isolation of HuMuSC

We have previously developed methods for isolation and purification functional satellite cells from human muscle tissue, and characterized the gene expression similarities and differences among different skeletal muscles [15,22]. This preparation of purified and minimally altered satellite cells enables in vitro and in vivo experimentation as well as translational applications and our prior work has shown remarkable similarity among HuMuSC from different muscles [11]. Immediately after surgical resection the muscle is stored in Collection media composed of 30% FBS in DMEM high glucose, supplemented with Pen Strep at 4°C. The human muscle biopsies corresponded to several types of muscles including: vastus lateralis, rectus abdominis, pectoralis, gracilis, gastrocnemius, latissimus, intercostal, gluteus, soleus, peroneal, tibialis posterior, tensor fascia lata and temporalis. The protocol for Human satellite cell activation begins 12 hrs before the start of processing the muscle biopsy, taking 2/3 of the muscle tissue specimen and storing at 4°C for the quiescent sample. The remaining 1/3 of the muscle biopsy is for the experimentally stimulated sample, and it is immediately macrodissociated: a mechanical dissociation of the muscle sample is done using a blade. To ensure a clear single cell suspension for Fluorescence-Activated Cell Sorting (FACS) we used enzymatic dissociation and filters as well as Magnetic Cell Separation (MACS LD) of hematopoietic cells (CD45+ and CD31+) from the cell suspension by means of antibodies and magnetic columns. To specifically sort MuSC, we used all the following antibodies as muscle stem cells surface markers: CD56, CD29 and CXCR4. Please refer to our detailed published protocol for all details [11,22]. Then the macrodissociated tissue is resuspended in 30 ml of Growth Media (Ham’s F10 basal media, 20% FBS, 1X Pen Strep, 1/500 1M HEPES, 5ng/ml FGF2) and transferred to a low attachment plate and incubated at 37°C for 12 hrs. After this incubation, the macrodissociated muscle tissue from the plate is transferred to a 50 ml conical tube, rinsed with 1x PBS, brought to volume 50 ml with 1x PBS, centrifuged at 2000 rpm for 5 min. The supernatant is aspirated and digestion media added and continued with the normal digestion/processing/staining protocol together with the quiescent muscle sample from the same individual [22]. The dates of samples were:

**Table:** 

Date of collection of the Samples	n = cells isolated by FACS
09/01/2016	16800
09/01/2016	10000
09/01/2016	12800
05/07/2017	4000
05/23/2017	56000
05/24/2017	30000
01/30/2019	26000
01/30/2019	26000
01/31/2019	16000
02/01/2019	30000
02/05/2019	10000
02/07/2019	15500
02/07/2019	10000
02/07/2019	25800
02/07/2019	10000
02/12/2019	59000
02/13/2019	15000
02/13/2019	150000
02/19/2019	9000
02/20/2019	15000
02/20/2019	10500
02/27/2019	8400
03/05/2019	17000
03/06/2019	20000
03/07/2019	15000
03/13/2019	60000
03/20/2019	14000
03/26/2019	12500
03/26/2019	5000
03/26/2019	26000
03/27/2019	6000
04/03/2019	9000
04/03/2019	300000
04/08/2019	24000
04/17/2019	38000
05/07/2019	12700
05/22/2019	4500
05/08/2019	6700
05/09/2019	23000
05/09/2019	39000
05/09/2019	20000
05/14/2019	3300
05/15/2019	110000
05/14/2019	4800
05/15/2019	5500
05/21/2019	7300
05/21/2019	4300
05/20/2019	5000
05/23/2019	10000
05/23/2019	36000
05/28/2019	8000
05/29/2019	11000
06/05/2019	180000
01/09/2020	18000
01/10/2020	50000
01/11/2020	70000
01/10/2020	50000
01/13/2020	8000
01/14/2020	11800
01/15/2020	80000
01/16/2020	18000
01/17/2020	400000
01/18/2020	10000
01/19/2020	28000
01/20/2020	28000
01/21/2020	40000
02/02/2021	37000
05/17/2021	5000
05/21/2021	45000
09/08/2021	25000
01/20/2022	18000
06/07/2022	60000
08/25/2022	60000
02/16/2023	50000
03/26/2023	105000
05/01/2023	6000

### EdU experiments

After the control and activated HuMuSC were isolated by FACS, they were plated in a 48 precoated plate with Matrigel, at a 10,000 cells per well and incubated at 37 degrees in grown media of 10% FBS in DMEM media plus 2 ul of EdU per 200 ul of media per well.After 3 days cells were fixed with PFA 4% and permeabilized with 10% NBF in PBS for 15 minutes. After EdU reaction (Click-iT Plus EdU Alexa Fluor 488 Imaging Kit catalog number: C10637) per manufacturer manual instructions, cells were counterstained with dapi and quantified in a Leica microscope to count number of positive cells over total cells per well. n = 3 paired control and activated HuMuSC. Grap Pad Prism v. 10, one way ANOVA, was used for statistical analysis.

### Single cell assay for transposase accessible chromatin (scATAC) in human satellite cells

All protocols to generate scATAC-seq data using the 10x Chromium platform, including sample preparation, library preparation and instrument and sequencing settings are published at the 10x genomics website.

To investigate the differentially open chromatin regions in controls quiescent vs. activated HuMuSC after the overnight activation model, right after the FACS isolation, the nuclei were isolated following guidelines for limited number and fragile cells by 10x Demonstrated protocol (Nuclei Isolation for Single Cell ATAC Sequencing CG00169 Rev E) and the nuclei sample from quiescent and activated HuMuSC were loaded into the chromium chip by the UCSF Genomic core for scATAC.

The scATAC-seq libraries were prepared and sequenced according to the Chromium Single Cell ATAC Reagent Kits User Guide (10x Genomics; CG000168 Rev B) and the BCL files subjected to the Cell Ranger pipeline. (Cell Ranger Software v.1.2.0;

**Data processing using Cell Ranger ATAC software 1.2.0** Cell Ranger and Loupe Cell Browser Version 5.0. were used as described before [39].

Analysis was done using R software [40], the Seurat [41] v4.3.0 and the Signac v1.9.0 packages [42], designed as a framework for the analysis of single-cell chromatin data and related vignettes [42]. When pre-processing chromatin data, Signac uses information from two related input files, both of which can be created using CellRanger:

- Peak/Cell matrix. This is analogous to the gene expression count matrix used to analyze single-cell RNA-seq. However, instead of genes, each row of the matrix represents a region of the genome (a peak), that is predicted to represent a region of open chromatin. Each value in the matrix represents the number of Tn5 integration sites for each single barcode (i.e., a cell) that map within each peak. You can find more detail on the 10X Website [42].
- Fragment file. This represents a full list of all unique fragments across all single cells. It is a substantially larger file, is slower to work with, and is stored on-disk (instead of in memory). However, the advantage of retaining this file is that it contains all fragments associated with each single cell, as opposed to only fragments that map to peaks. More information about the fragment file can be found on the 10x Genomics website or on the sinto website [42].

Single-cell chromatin state analysis with Signac [42] was employed to enable end-to-end analysis of chromatin data and includes functionality for diverse analysis tasks, including identifying cells from background noncell-containing barcodes, calling peaks, quantifying counts in genomic regions, quality control filtering of cells, dimension reduction, clustering, integration with single-cell gene expression data, interactive genome browser-style data visualization, finding differentially accessible peaks, finding enriched DNA sequence motifs, transcription factor foot printing and linking peaks to potential regulatory target genes [43]. The goal of quality control is to keep only high quality cells (i.e., remove low quality cells (dead or dying cells), cell-free RNA, or doublets). We computed per-cell quality control (QC) metrics using the DNA accessibility assay, including the strength of the nucleosome banding pattern and transcriptional start site (TSS) enrichment score and removed low-quality cells based on these QC metrics resulting in a dataset of 9,232 cells distributed in 7,850 from activated HuMuSC and 2,411 from the control HuMuSC [43].

### Computing QC Metrics

Nucleosome banding pattern: The histogram of DNA fragment sizes (determined from the paired-end sequencing reads) was examined for a strong nucleosome banding pattern. This was calculated per single cell, and used to quantify the approximate ratio of mononucleosomal to nucleosome-free fragments (stored as nucleosome_signal).

Transcriptional start site (TSS) enrichment score: The ENCODE project has defined an ATAC-seq targeting score based on the ratio of fragments centered at the TSS to fragments in TSS-flanking regions. Poor ATAC-seq experiments typically will have a low TSS enrichment score. This metric was used for each cell with the TSSEnrichment function, and the results are stored in metadata under the column name TSS.enrichment.

Total number of fragments in peaks: Cells with very few reads were excluded due to low sequencing depth. Cells with extremely high levels may represent doublets, nuclei clumps, or other artefacts.

Fraction of fragments in peaks: Represents the fraction of all fragments that fall within ATAC-seq peaks. Cells with low values (i.e., < 15–20%) were removed.

We compared QC control with SCTransform method and with log normalization, obtaining 6 clusters with the first method and 5 with log normalization, very comparable otherwise. Non-linear dimension reduction and clustering we looked at some different values of resolution in clustering and chose resolution = 0.8 (default).

Next, we processed the gene expression assay by normalizing RNA counts with SCTransform (p < 0.05) and Seurat. Significant peaks (p < 0.05) allowed for gene enrichment identification and pathway analysis using the Reactome PA [44] and WikiPathways [38].

### Cytokine autoantibody panels

HuMuSC from one Vastus lateralis, one Pectoralis and one Rectus muscles from humans ages 29, 31 and 44 years old distributed in three samples with 1 duplicates each (n = 3). were cryopreserved and were later thawed and plated on Matrigel precoated dishes in growth media (20% FBS in DMEM high glucose + P/S). After a media change 12 hrs later, HuMuSC were cultured under regular conditions for 3–6 days. Then the supernatant and the HuMuSC were harvested in separate samples and sent to Eve Technologies according to their protocol for sample collection [45]. Undiluted supernatant and cell lysate samples were profiled for 83 different human cytokines using the Human Cytokine Array/Chemokine Array 71-Plex Panel and the 12-plex featured assay (catalog no: HD71; Eve Technologies). The controls included growth media and lysis buffer processed under the same conditions as the samples but without cells. Samples were prepared according to Eve Technologies guidelines for the MILLIPLEX Human Cytokine Autoantibody Panels and the magnetic microsphere beads from Luminex Corp. Briefly, each set of beads is distinguished by different ratios of two internal dyes yielding a unique fluorescent signature to each bead set. Capture antibodies or antigens were coupled to the magnetic beads. Eve Technology results were based on the fluorescence intensity values in direct proportion to the standard known concentrations to calculate the concentration of cytokine proteins in our samples. Fluorescence values of the control supernatant or the control lysis buffer were subtracted from the fluorescence values of the supernatant sample or HuMuSC samples respectively after correction negative values were expressed as minus X and zero values as 0.

### RT-QPCR

Total RNA was isolated using the RNAeasy isolation kit (Qiagen Cat # 74104). RNA was transcribed into cDNA with High-Capacity cDNA Reverse Transcription kit (ThermoFisher Scientific, Cat # 4368814). cDNA was then pre-amplified with GE PreAmp Master Mix (Fluidigm Inc, Cat#100-5876C2). Real-time quantitative PCR was performed in triplicated with either Taqman Universal PCR Master Mix (Life Technologies) or SybrGreen primers on a Viia7 thermocycler (Life Technologies). Taqman primers are listed in the Table 1 below. B-Actin, RPS-13 and GAPDH were used for normalization as endogenous control. Primers included were from Thermofisher scientific and the sequences of the primers pairs related to each gene of these reference sequences can be found on the National Center for Biotechnology Information (NCBI) Primer Blast page.

**Table 1:** List of primers used in quantitative RT-PCR.

Name	Thermofisher Primer Reference #	NCBI RefSeq mRNA Accession number
**CXCL14**	Hs01557413	NM_002982.4
**CCL2**	Hs00234140	NM_002982
**CXCL1**	Hs00236937	NP_001502.1
**CXCL8**	Hs00608272_m1	NM_000584.4
**TNFRSF12A**	Hs00171993_m1	NM_012260.4
**CSF-1**	Hs00911250_m1	NM_000757.4
**FGF2**	Hs04234540_g1	NM_002006.6
**PAX7**	Hs00242962	NM_001368930.1
**MYOD**	Hs00159528	NM_002478.5
**RPS-13**	Hs01011487_g1	NM_001017
**B-ACTIN**	Hs0106665	NM_001101.4
**GAPDH**	Hs02758991	NM_002046.3

To analyze RT-PCR data, Wilcoxon tests in Prism software were performed on 3–6 paired control and activated HuMuSC (n = 3–6), each pair coming from one human donor muscle either vastus lateralis or rectus, p < 0.05 as significant.

### Xenotransplants

For surgical procedures, animals were anesthetized with isoflurane and appropriate anesthesia is determined by paw pinch. One dose of NSAID and one dose of buprenorphine were administered at the conclusion of the procedure after the animal was removed from anesthesia. Mice were placed on a heating pad until ambulatory. The animals were assessed for pain and analgesics buprenorphine and non-steroidal anti-inflammatory agents were provided every 12−24 hours as needed based on pain score. Mice in all experiments were euthanized by carbon dioxide inhalation and cervical dislocation. 2,000 HuMuSC were co-injected with Bupivacaine in the left TA of irradiated NSG mice (6−10 weeks old). To test the effects of cytokine’s on engraftment and myofiber formation of HuMuSC, we treated with a daily dose during 7 days of either, a CCL2 antagonist (Bindarit -the selective inhibitor of CCL2, CCL7 and CCL8), or the Recombinant human CCL-2 from Peprotech 100 mg/kg, or vehicle only controls (n = 10 mice per group). Three weeks after HuMuSC transplantation, animals were sacrificed, and the TA was processed for Cryosections and immunostaining. Controls were the same vehicle, volume, administration route (IP) and frequency as the Bindarit group but without the Bindarit agent. In our experiment we used Bindarit at 100 mg/kg IP BID. Three weeks after transplantation the TAs were collected in OCT, cryopreserved and cryosected. The number and diameter of the human fibers Dystrophin positive in the mouse muscle niche, as well as the HuMuSC expressing PAX7 positive Human Spectrin positive immunostaining were quantified and compared using in Graph pad prism v 10, unpaired t test, p < 0.05 as significant.

Using three independent donors (n based on similarity of HuMuSC phenotype and function in different individuals) [15,22]. from which HuMuSC were isolated in independent xenotransplant experiments, 2,000 HuMuSC were co-injected with Bupivacaine in the left TA of NSG mice (6–10 weeks old). Three weeks after HuMuSC transplantation, animals were euthanized, and the TA was processed for cryosections and immunostaining.

### Analysis of images in Fiji

Images were captured under the same conditions under the same magnificaiton for accurate comparison. Regions of Interest (ROIa) were defined and the Coloc 2 tool was used to calculate colocalization metrics. We determined the significance of colocalization findings based on the Pearson correlation. In this way we compared multi-fluorescence imaging followed by Object-based colocalization analysis tools [46].

### Ethics

**Human subjects:** This study was conducted under the approval of the Institutional Review Board at The University of California San Francisco (UCSF), IRB Number: 11-07323l, continuously active through the entire study time period. Written informed consent was obtained from all subjects.

**Animal experimentation:** All procedures were approved and performed in accordance with the UCSF Institutional Animal Care and Use Committee (Protocols #181101).

## Supporting information

S1 File Muscle study approval letters. (ZIP)
