# Supplementary material for: Control of quiescence and activation of human muscle stem cells by cytokines
Source: PLoS One. 2025 Dec 5;20(12):e0327701. doi: 10.1371/journal.pone.0327701 (PMC12680340; doi:10.1371/journal.pone.0327701)
Supplement: S1 File — (ZIP) [file pone.0327701.s001.zip › muscle study approval letters/Outcome_Letter15.pdf]

UCSF
University of California
San Francisco

irb.ucsf.edu

Human Research Protection Program
Institutional Review Board (IRB)

Expedited Review Approval

Principal Investigator
Dr. Jason Pomerantz, MD

Type of Submission: Continuing Review Submission Form
Study Title: Collection of human skeletal muscle cells to study cellular mechanisms of muscle regeneration
Study Status: Expedited GESCR/Expedited IRB

IRB #: 11-07323
Reference #: 338776
Committee of Record: San Francisco General Hospital Panel
Study Risk Assignment: Minimal

Approval Date: 03/02/2022 **Expiration Date:** 03/01/2023

Regulatory Determinations Pertaining to this Approval:

This research satisfies the following condition(s) for the involvement of children:
45 CFR 46.404, 21 CFR 50.51: Research not involving greater than minimal risk.

Parental Permission and Assent:
The permission of one parent or guardian is sufficient.
The assent of the children will be obtained.

The research meets conditions of 45 CFR 46.205 for the involvement of neonates.

Individual Research HIPAA Authorization is required of all subjects. Use the Permission to Use Personal Health Information for Research form, the Authorization for Use and Release of Individually Identifiable Health Information Collected for VHA Research (if the VA is a site) or the local HIPAA authorization for research form, if the study involves access to patient records outside UCSF.

A waiver of HIPAA Authorization and consent is acceptable for the recruitment procedures to identify potential subjects. The recruitment procedures involve routine review of medical or other records, do not adversely affect the rights and welfare of the individuals, and pose minimal risk to subjects and their privacy, based on, at least, the presence of the following elements: (1) an adequate plan to protect the identifiers from improper use and disclosure; (2) an adequate plan to destroy the identifiers at the earliest opportunity consistent with conduct of the research, or a

health or research justification for retaining the identifiers was provided or such retention is otherwise required by law; (3) adequate written assurances that the requested information will not be reused or disclosed to any other person or entity, except as required by law, for authorized oversight of the research study, or for other research for which the use or disclosure of the requested information would be permitted by the Privacy Rule; (4) the research could not practicably be conducted without the waiver; and (5) study recruitment could not practicably be conducted without access to and use of the requested information. The research subjects will sign a consent form prior to participation in the study.

This submission was eligible for expedited review as:

Category 9: Continuing review of research, not conducted under an IND or IDE where categories two (2) through eight (8) do not apply but the IRB has determined and documented at a convened meeting that the research involves no greater than minimal risk and no additional risks have been identified

IRB Comments:

Important Information for the Principal Investigator (updated 2/27/2022):

- It is the Principal Investigator's responsibility to ensure that all study personnel are properly trained for their roles in the study.

- It is the Principal Investigator's responsibility to ensure that the list of personnel in the IRB application is current and those listed as Key Study Personnel maintain current CITI Human Subjects Protection Training. Information about CITI Human Subjects Training can be found on the IRB website.

- It is the Principal Investigator's responsibility to report to the IRB any protocol violations, adverse events and other reportable events/items that meet the UCSF reporting requirements. Please review and follow the Post-Approval Reporting Requirements Summary Sheet.

- If this study has collaborating sites with their own reviewing IRBs, it is the Principal Investigator's responsibility to ensure that the collaborating sites have current IRB approval prior to engaging with them in any research activities, including sharing UCSF data or samples with them.

- If the IRB has approved the study to enroll non-English speaking participants, the Principal Investigator must ensure that the consent method follows current UCSF guidelines based on the IRB-approved method (Short Form Method vs. Preferred Method).

- The UCSF IRB strongly encourages enrollment of diverse participants. UCSF's Clinical & Translational Science Institute (CTSI) Participant Recruitment Program and the CTSI Integrating Special Populations Core offer tools, services, and consultations to support recruitment of underrepresented populations. For more information and to request a consultation, see: recruit.ucsf.edu

- A Data Security Risk Assessment by UCSF IT **AND** Data Sharing Review by the Office of Sponsored Research (OSR -- Industry Contracts Division) must be completed if your study involves: **the collection, transmission, or storage of information when that data will be shared with or be accessible to any non-UCSF entity (e.g., pharmaceutical companies, NIH, UCSF Affiliated Institutions) or individual.** This includes the use of third-party or vendor-hosted applications. UCSF or department-hosted applications may also need to be assessed,

particularly if they are new applications or have never been reviewed by UCSF IT Security.

- Third-party or vendor-hosted applications include cloud-hosted applications and applications hosted by collaborating institutions
- UCSF or department-hosted applications include any application managed by UCSF or developed by the department

These requirements apply for both identifiable and de-identified data for funded and unfunded research. **Questions about these policies must be directed to UCSF IT and OSR, not to the IRB.**

- To determine if a Data Security Risk Assessment is required, contact the intake team at datasecurity@ucsf.edu. More information about the assessment process is available at https://it.ucsf.edu/service/it-security-risk-assessment

- For information about Data Sharing Review: https://data.ucsf.edu/edrp and https://icd.ucsf.edu/material-transfer-and-data-agreements

All changes to a study must receive UCSF IRB approval before they are implemented. Follow the modification request instructions. The only exception to the requirement for prior UCSF IRB review and approval is when the changes are necessary to eliminate apparent immediate hazards to the subject (45 CFR 46.103.b.4, 21 CFR 56.108.a). In such cases, report the actions taken by submitting a Protocol Violation/Incident Report in iRIS. (There is an option under Major Research-Related Incident -- Change in protocol necessary to immediately protect research participants or others.) If the change will be a permanent change to the study, you must also submit a Modification form.

Conducting Research During the COVID-19 Public Health Outbreak: Please visit the Interim UCSF Policy on Human Subjects-Related Research Visits at San Francisco Campuses during COVID-19 Outbreak, which can be found at https://research.ucsf.edu/guidance-onsite-clinical-research-activities to determine whether and how this Policy may affect this IRB approved or exempt study.

Expiration Notice: The iRIS system will generate an email notification eight weeks prior to the expiration of this study's approval. However, it is your responsibility to ensure that an application for continuing review approval has been submitted by the required time.

In addition, you are required to submit a study closeout report at the completion of the project.

Documents Reviewed and Approved with this Submission:

Consent Documents

Study Consent Form			
Title	Version #	Version Date	Outcome
Parent consent for blood draw portion2016	Version 1.8	02/25/2018	Approved
ConsentDocument for blood draw portion 2016	Version 1.10	02/04/2018	Approved
AssentDocument including blood draw 2016	Version 1.6	02/04/2018	Approved
Parent consent	Version 1.22	02/25/2018	Approved

Collection of human skeletal muscle cells assent for ages 7-12	Version 1.10	02/23/2018	Approved
Collection of human skeletal muscle cells to study cellular mechanisms of muscle regeneration.	Version 1.18	02/25/2018	Approved

For a list of all currently approved documents, follow these steps: Go to My Studies and open the study – Click on Informed Consent to obtain a list of approved consent documents and Other Study Documents for a list of other approved documents.

San Francisco Veterans Affairs Medical Center (SFVAMC): If the SFVAMC is engaged in this research, you must secure approval of the VA Research & Development Committee in addition to UCSF IRB approval and follow all applicable VA and other federal requirements. The IRB website has more information.
