# Supplementary material for: Control of quiescence and activation of human muscle stem cells by cytokines
Source: PLoS One. 2025 Dec 5;20(12):e0327701. doi: 10.1371/journal.pone.0327701 (PMC12680340; doi:10.1371/journal.pone.0327701)
Supplement: S1 File — (ZIP) [file pone.0327701.s001.zip › muscle study approval letters/Outcome_Letter17.pdf]

HUMAN GAMETE, EMBRYO AND STEM CELL RESEARCH COMMITTEE
HUMAN RESEARCH PROTECTION PROGRAM, Box 0962
UNIVERSITY OF CALIFORNIA, SAN FRANCISCO
415-476-1814
http://irb.ucsf.edu/stem-cell

HUMAN GAMETE, EMBRYO AND STEM CELL RESEARCH COMMITTEE DETERMINATION LETTER

TO: Dr. Jason Pomerantz, MD
PROJECT TITLE: Collection of human skeletal muscle cells to study cellular mechanisms of muscle regeneration
STUDY NUMBER: 11-07323
DATE: 03/07/2024
STUDY SPONSOR: Glenn Foundation For Medical Research, NIH Natl Inst Arthr, Musculoskel & Skin, Plastic Surgery Educational Foundation
FUNDING REFERENCE NUMBER: P0532938, P0521837, P0512852
APPLICATION TYPE: Continuing Review Submission Form
REVIEW LEVEL: Expedite

Outcome
This letter certifies that on 03/07/2024 this application was approved by the Human Gamete, Embryo and Stem Cell Research (GESCR) Committee. The GESCR Committee functions as the University of California, San Francisco Stem Cell Research Oversight (SCRO) Committee.

This was carried out by an Expedited review process because the submission is a Continuing Review of a currently approved protocol with no changes.

Comments
- If materials are shared with other researchers, the GESCR Committee recommends a Material Transfer Agreement (MTA) including any research restrictions stated in the consent forms. (e.g. No reproductive research or human reproductive cloning).

Approved Human Embryonic Stem Cell (hESC) Lines
N/A

Period of Approval
GESCR approval is valid from **03/07/2024** through **03/06/2025**.
This study must be renewed by the expiration date if work is to continue. Also, prior GESCR approval is required before implementing any changes in the protocol.

Additional Review(s)
This protocol requires approval by the:
- Institutional Review Board (IRB)
- Institutional Animal Care and Use Committee (IACUC)

Research Category

In particular, this protocol involves:

- The identities of the tissue/cell donors are known to the principal investigator.
- The injection/transplantation of adult skeletal muscle stem cells into the skeletal muscles of the legs and head of immunodeficient mouse and rat models to examine regenerative function.

Sincerely,

Marcelle I Cedars, M.D.
Chair, Human Gamete Embryo and Stem Cell Research Committee
