# Supplementary material for: Control of quiescence and activation of human muscle stem cells by cytokines
Source: PLoS One. 2025 Dec 5;20(12):e0327701. doi: 10.1371/journal.pone.0327701 (PMC12680340; doi:10.1371/journal.pone.0327701)
Supplement: S1 File — (ZIP) [file pone.0327701.s001.zip › muscle study approval letters/Outcome_Letter12.pdf]

UCSF
University of California
San Francisco

Human Research Protection Program
Institutional Review Board (IRB)

Expedited Review Approval

Principal Investigator
Dr. Jason Pomerantz MD, MD

Type of Submission: Continuing Review Submission Form
Study Title: Collection of human skeletal muscle cells to study cellular mechanisms of muscle regeneration

IRB #: 11-07323
Reference #: 243952
Committee of Record: San Francisco General Hospital Panel
Study Risk Assignment: Minimal

Approval Date: 03/09/2019 **Expiration Date:** 03/08/2020

IRB Comments:
The UCSF IRB records indicate that **Emily Barruet, Katharine Pomerantz and Jaclyn Cusano** need to complete or renew CITI Human Subjects Protection Training. This Key Study Personnel cannot participate in this research study or any other UCSF research study involving human subjects research until the CITI Human Subjects Protection Training is completed or renewed. All UCSF Key Study Personnel conducting human research must complete CITI Human Subjects Protection Training. It is the Principal Investigator's responsibility to ensure that all Key Study Personnel complete and maintain CITI Human Subjects Protection Training.
Training records in CITI and iRIS are linked by email address. If this notice seems in error, please make sure the email address in CITI matches the email address used for iRIS. In iRIS, the email address is found in the "My Account Information" section. For more information about CITI, including a link to the CITI completion list and the definition of Key Study Personnel, please visit the IRB's CITI Human Subjects Protection Training page: http://irb.ucsf.edu/citi-human-subjects-training.

Regulatory Determinations Pertaining to this Approval:

This research satisfies the following condition(s) for the involvement of children:
45 CFR 46.404, 21 CFR 50.51: Research not involving greater than minimal risk.

Parental Permission and Assent:
The permission of one parent or guardian is sufficient.
The assent of the children will be obtained.

The research meets conditions of 45 CFR 46.205 for the involvement of neonates.

Individual Research HIPAA Authorization is required of all subjects. Use the Permission to Use

Personal Health Information for Research form.

Individual Research HIPAA Authorization is required of all subjects. Use the Permission to Use Personal Health Information for Research form. A waiver of HIPAA Authorization and consent is acceptable for the recruitment procedures to identify potential subjects. The recruitment procedures involve routine review of medical or other records, do not adversely affect the rights and welfare of the individuals, and pose minimal risk to subjects and their privacy, based on, at least, the presence of the following elements: (1) an adequate plan to protect the identifiers from improper use and disclosure; (2) an adequate plan to destroy the identifiers at the earliest opportunity consistent with conduct of the research, or a health or research justification for retaining the identifiers was provided or such retention is otherwise required by law; (3) adequate written assurances that the requested information will not be reused or disclosed to any other person or entity, except as required by law, for authorized oversight of the research study, or for other research for which the use or disclosure of the requested information would be permitted by the Privacy Rule; (4) the research could not practicably be conducted without the waiver; and (5) study recruitment could not practicably be conducted without access to and use of the requested information. The research subjects will sign a consent form prior to participation in the study.

This submission was eligible for expedited review as:
Category 9: Renewal of other minimal risk research protocols: Continuing review of research, not conducted under an IND or IDE where categories 2 through 8 do not apply but the IRB has determined and documented at a convened meeting that the research involves no greater than minimal risk and no additional risks have been identified

All changes to a study must receive UCSF IRB approval before they are implemented. Follow the modification request instructions. The only exception to the requirement for prior UCSF IRB review and approval is when the changes are necessary to eliminate apparent immediate hazards to the subject (45 CFR 46.103.b.4, 21 CFR 56.108.a). In such cases, report the actions taken by following these instructions.

Expiration Notice: The iRIS system will generate an email notification eight weeks prior to the expiration of this study's approval. However, it is your responsibility to ensure that an application for continuing review approval has been submitted by the required time. In addition, you are required to submit a study closeout report at the completion of the project.

Documents Reviewed and Approved with this Submission:

Consent Documents

Study Consent Form			
Title	Version #	Version Date	Outcome
Parent consent for blood draw portion2016	Version 1.5	02/25/2018	Approved
ConsentDocument for blood draw portion 2016	Version 1.7	02/04/2018	Approved

AssentDocument including blood draw 2016	Version 1.3	02/04/2018	Approved
Parent consent	Version 1.18	02/25/2018	Approved
Collection of human skeletal muscle cells assent for ages 7-12	Version 1.7	02/23/2018	Approved
Collection of human skeletal muscle cells to study cellular mechanisms of muscle regeneration.	Version 1.15	02/25/2018	Approved

For a list of all currently approved documents, follow these steps: Go to My Studies and open the study – Click on Informed Consent to obtain a list of approved consent documents and Other Study Documents for a list of other approved documents.

San Francisco Veterans Affairs Medical Center (SFVAMC): If the SFVAMC is engaged in this research, you must secure approval of the VA Research & Development Committee in addition to UCSF IRB approval and follow all applicable VA and other federal requirements. The UCSF IRB website has more information.
