# Supplementary material for: Control of quiescence and activation of human muscle stem cells by cytokines
Source: PLoS One. 2025 Dec 5;20(12):e0327701. doi: 10.1371/journal.pone.0327701 (PMC12680340; doi:10.1371/journal.pone.0327701)
Supplement: S1 File — (ZIP) [file pone.0327701.s001.zip › muscle study approval letters/Outcome_Letter10.pdf]

University of California
San Francisco

Human Research Protection Program
Institutional Review Board (IRB)

Expedited Review Approval

Principal Investigator
Dr. Jason Pomerantz MD, MD

Type of Submission: Modification Form
Study Title: Collection of human skeletal muscle cells to study cellular mechanisms of muscle regeneration

IRB #: 11-07323
Reference #: 202979
Committee of Record: San Francisco General Hospital Panel
Study Risk Assignment: Minimal

Approval Date: 10/17/2017 **Expiration Date:** 03/27/2018

IRB Comments:

All changes to a study must receive UCSF IRB approval before they are implemented. Follow the modification request instructions. The only exception to the requirement for prior UCSF IRB review and approval is when the changes are necessary to eliminate apparent immediate hazards to the subject (45 CFR 46.103.b.4, 21 CFR 56.108.a). In such cases, report the actions taken by following these instructions.

Expiration Notice: The iRIS system will generate an email notification eight weeks prior to the expiration of this study's approval. However, it is your responsibility to ensure that an application for continuing review approval has been submitted by the required time. In addition, you are required to submit a study closeout report at the completion of the project.

Documents Reviewed and Approved with this Submission:

Consent Documents

Study Consent Form			
Title	Version #	Version Date	Outcome
Parent consent for blood draw portion2016	Version 1.3	02/25/2016	Approved
ConsentDocument for blood draw portion 2016	Version 1.5	02/04/2016	Approved
Parent consent	Version 1.16	02/25/2016	Approved
Collection of human skeletal muscle cells to	Version 1.13	02/25/2016	Approved

study cellular mechanisms of muscle regeneration.			

Other Study Documents

Study Document			
Title	Version #	Version Date	Outcome
Protection_of_Human_Subjects Pomerantz R01 1-30	Version 1.0	09/26/2017	Approved
Research_Strategy_Pomerantz R01_2-3FINAL	Version 1.0	09/26/2017	Approved

For a list of all currently approved documents, follow these steps: Go to My Studies and open the study – Click on Informed Consent to obtain a list of approved consent documents and Other Study Documents for a list of other approved documents.

San Francisco Veterans Affairs Medical Center (SFVAMC): If the SFVAMC is engaged in this research, you must secure approval of the VA Research & Development Committee in addition to UCSF IRB approval and follow all applicable VA and other federal requirements. The UCSF IRB website has more information.
