# Supplementary material for: Control of quiescence and activation of human muscle stem cells by cytokines
Source: PLoS One. 2025 Dec 5;20(12):e0327701. doi: 10.1371/journal.pone.0327701 (PMC12680340; doi:10.1371/journal.pone.0327701)
Supplement: S1 File — (ZIP) [file pone.0327701.s001.zip › muscle study approval letters/Outcome_Letter.pdf]

UCSF
University of California
San Francisco

Human Research Protection Program
Committee on Human Research

Notification of Full Committee Approval

Principal Investigator
Jason H Pomerantz

Co-Principal Investigator

Type of Submission: Initial Review Submission Packet
Study Title: Collection of human skeletal muscle cells to study cellular mechanisms of muscle regeneration

IRB #: 11-07323
Reference #: 029413

Reviewing Committee: San Francisco General Hospital Panel

Study Risk Assignment: Minimal

Approval Date: 03/29/2012 **Expiration Date:** 03/28/2013

Regulatory Determinations Pertaining to This Approval:

This research satisfies the following condition(s) for the involvement of children:

45 CFR 46.404, 21 CFR 50.51: Research not involving greater than minimal risk.

Parental Permission and Assent:
The permission of one parent or guardian is sufficient.
The assent of the children will be obtained.

Individual HIPAA authorization is required.
A waiver of HIPAA Authorization is acceptable for the recruitment procedures to identify potential subjects. The recruitment procedures involve routine review of medical or other records, do not adversely affect the rights and welfare of the individuals, and pose minimal risk to their privacy, based on, at least, the presence of the following elements: (1) an adequate plan to protect the identifiers from improper use and disclosure; (2) an adequate plan to destroy the identifiers at the earliest opportunity consistent with conduct of the research, or a health or research justification for retaining the identifiers was provided or such retention is otherwise required by law; (3) adequate written assurances that the requested information will not be reused or disclosed to any other person or entity, except as required by law, for authorized oversight of the research study, or for other research for which the use or disclosure of the requested information would be permitted by the Privacy Rule; (4) the research could not practicably be conducted without the waiver; and (5) the research could not practicably be conducted without access to and use of the requested information.

IRB Comments (if applicable):

All changes to a study must receive CHR approval before they are implemented. Follow the modification request instructions. The only exception to the requirement for prior CHR review and approval is when the changes are necessary to eliminate apparent immediate hazards to the subject (45 CFR 46.103.b.4, 21 CFR 56.108.a). In such cases, report the actions taken by following these instructions.

Expiration Notice: The iMedRIS system will generate an email notification eight weeks prior to the expiration of this study's approval. However, it is your responsibility to ensure that an application for continuing review approval has been submitted by the required time. In addition, you are required to submit a study closeout report at the completion of the project.

Approved Documents: To obtain a list of documents that were approved with this submission, follow these steps: Go to My Studies and open the study – Click on Submissions History – Go to Completed Submissions – Locate this submission and click on the Details button to view a list of submitted documents and their outcomes.

For a list of all currently approved documents, follow these steps: Go to My Studies and open the study – Click on Informed Consent to obtain a list of approved consent documents and Other Study Documents for a list of other approved documents.

San Francisco Veterans Affairs Medical Center (SFVAMC): If the SFVAMC is engaged in this research, you must secure approval of the VA Research & Development Committee in addition to CHR approval and follow all applicable VA and other federal requirements. The CHR website has more information.
