# Supplementary material for: Control of quiescence and activation of human muscle stem cells by cytokines
Source: PLoS One. 2025 Dec 5;20(12):e0327701. doi: 10.1371/journal.pone.0327701 (PMC12680340; doi:10.1371/journal.pone.0327701)
Supplement: S1 File — (ZIP) [file pone.0327701.s001.zip › muscle study approval letters/GESCR Outcome_Letter.pdf]

HUMAN GAMETE, EMBRYO AND STEM CELL RESEARCH COMMITTEE
HUMAN RESEARCH PROTECTION PROGRAM, Box 0962
UNIVERSITY OF CALIFORNIA, SAN FRANCISCO
(415) 476-9134
http://www.research.ucsf.edu/sc

HUMAN GAMETE, EMBRYO AND STEM CELL RESEARCH COMMITTEE DETERMINATION LETTER

TO: Jason H Pomerantz

PROJECT TITLE: Collection of human skeletal muscle cells to study cellular mechanisms of muscle regeneration

STUDY NUMBER: 11-07323

DATE: 01/03/2013

STUDY SPONSOR: CALIF Inst for Regenerative Medicine

AWARD REFERENCE NUMBER: P0051512

APPLICATION TYPE: Submission Correction for Modification Form

REVIEW LEVEL: Expedite

Outcome

This letter certifies that on 01/03/2013 this application was approved by the Human Gamete, Embryo and Stem Cell Research (GESCR) Committee. The GESCR Committee functions as the University of California, San Francisco Stem Cell Research Oversight (SCRO) Committee. This was carried out by an Expedited review process because the protocol involves the study of adult muscle stem cells in vitro and in non-primate animals. The only modification in this submission is the addition of new CIRM funding.

Comments

- If materials are shared with other researchers, the GESCR Committee recommends a Material Transfer Agreement (MTA) including any research restrictions stated in the consent forms. (e.g. No reproductive research or human reproductive cloning).

Approved Human Embryonic Stem Cell (hESC) Lines

The GESCR Committee approved the use of the following hESC lines for this protocol: N/A

Period of Approval

GESCR approval is valid from **01/03/2013** through **01/02/2014**.
This study must be renewed by the expiration date if work is to continue. Also, prior GESCR approval is required before implementing any changes in the protocol.

Additional Review(s)

This protocol requires approval by the:

- Committee on Human Research (CHR).
- Institutional Animal Care Use Committee (IACUC).

Research Category

In particular, this protocol involves:

- The identities of the tissue/cell donors are known to the principal investigator
- The injection/Transplantation of adult skeletal muscle stem cells into the skeletal muscles of the legs and head of immunodeficient mouse and rat models to examine regenerative function.

Sincerely,

Richard M. Wagner, MA, CIP, CCRP
Regulatory Reviewer, Human Gamete Embryo and Stem Cell Research Committee
Associate Director, Human Research Protection Program

Marcelle I Cedars, M.D.
Chair, Human Gamete Embryo and Stem Cell Research Committee
