# Supplementary material for: Control of quiescence and activation of human muscle stem cells by cytokines
Source: PLoS One. 2025 Dec 5;20(12):e0327701. doi: 10.1371/journal.pone.0327701 (PMC12680340; doi:10.1371/journal.pone.0327701)
Supplement: S1 File — (ZIP) [file pone.0327701.s001.zip › muscle study approval letters/IRB Outcome_Letter.pdf]

UCSF
University of California
San Francisco

Human Research Protection Program
Committee on Human Research

Notification of Expedited Review Approval

Principal Investigator
Jason H Pomerantz

Co-Principal Investigator

Type of Submission: Modification Form
Study Title: Collection of human skeletal muscle cells to study cellular mechanisms of muscle regeneration

IRB #: 11-07323
Reference #: 059638
Committee of Record: San Francisco General Hospital Panel
Study Risk Assignment: Minimal

Approval Date: 1/3/2013 **Expiration Date:** 03/28/2013

All changes to a study must receive CHR approval before they are implemented. Follow the modification request instructions. The only exception to the requirement for prior CHR review and approval is when the changes are necessary to eliminate apparent immediate hazards to the subject (45 CFR 46.103.b.4, 21 CFR 56.108.a). In such cases, report the actions taken by following these instructions.

Expiration Notice: The iMedRIS system will generate an email notification eight weeks prior to the expiration of this study's approval. However, it is your responsibility to ensure that an application for continuing review approval has been submitted by the required time. In addition, you are required to submit a study closeout report at the completion of the project.

Approved Documents: To obtain a list of documents that were approved with this submission, follow these steps: Go to My Studies and open the study – Click on Submissions History – Go to Completed Submissions – Locate this submission and click on the Details button to view a list of submitted documents and their outcomes.

For a list of all currently approved documents, follow these steps: Go to My Studies and open the study – Click on Informed Consent to obtain a list of approved consent documents and Other Study Documents for a list of other approved documents.

San Francisco Veterans Affairs Medical Center (SFVAMC): If the SFVAMC is engaged in this research, you must secure approval of the VA Research & Development Committee in addition to CHR approval and follow all applicable VA and other federal requirements. The CHR website has more information.
